# Toxoplasma gondii AP2XII-2 Contributes to Proper Progression through S-Phase of the Cell Cycle

**DOI:** 10.1128/mSphere.00542-20

**Published:** 2020-09-16

**Authors:** Sandeep Srivastava, Michael W. White, William J. Sullivan

**Affiliations:** a Department of Pharmacology & Toxicology, Indiana University School of Medicine, Indianapolis, Indiana, USA; b Department of Microbiology & Immunology, Indiana University School of Medicine, Indianapolis, Indiana, USA; c Department of Global Health, University of South Florida, Tampa, Florida, USA; University at Buffalo

**Keywords:** *Apicomplexa*, *Toxoplasma*, cell cycle, chromatin, differentiation, parasites, transcription

## Abstract

Toxoplasma gondii is a protozoan parasite that causes lifelong chronic infection that can reactivate in immunocompromised individuals. Upon infection, the replicative stage (tachyzoite) converts into a latent tissue cyst stage (bradyzoite). Like other apicomplexans, T. gondii possesses an extensive lineage of proteins called ApiAP2s that contain DNA-binding domains first characterized in plants. The function of most ApiAP2s is unknown. We previously found that AP2IX-4 is a cell cycle-regulated ApiAP2 expressed only in dividing parasites as a putative transcriptional repressor. In this study, we purified proteins interacting with AP2IX-4, finding it to be a component of the recently characterized microrchidia (MORC) transcriptional repressor complex. We further analyzed AP2XII-2, another cell cycle-regulated factor that associates with AP2IX-4. We monitored parallel expression of AP2IX-4 and AP2XII-2 proteins in tachyzoites, detecting peak expression during S/M phase. Unlike AP2IX-4, which is dispensable in tachyzoites, loss of AP2XII-2 resulted in a slowed tachyzoite growth due to a delay in S-phase progression. We also found that AP2XII-2 depletion increased the frequency of bradyzoite differentiation *in vitro*. These results suggest that multiple AP2 factors collaborate to ensure proper cell cycle progression and tissue cyst formation in T. gondii.

**IMPORTANCE** Toxoplasma gondii is a single-celled parasite that persists in its host by converting into a latent cyst stage. This work describes a new transcriptional factor called AP2XII-2 that plays a role in properly maintaining the growth rate of replicating parasites, which contributes to signals required for development into its dormant stage. Without AP2XII-2, *Toxoplasma* parasites experience a delay in their cell cycle that increases the frequency of latent cyst formation. In addition, we found that AP2XII-2 operates in a multisubunit complex with other AP2 factors and chromatin remodeling machinery that represses gene expression. These findings add to our understanding of how *Toxoplasma* parasites balance replication and dormancy, revealing novel points of potential therapeutic intervention to disrupt this clinically relevant process.

## Abstract (precis)

Toxoplasma gondii is a single-celled parasite that persists in its host by converting into a latent cyst stage. This work describes a new transcriptional factor called AP2XII-2 that plays a role in properly maintaining the growth rate of replicating parasites, which contributes to signals required for development into its dormant stage. Without AP2XII-2, *Toxoplasma* parasites experience a delay in their cell cycle that increases the frequency of latent cyst formation. In addition, we found that AP2XII-2 operates in a multisubunit complex with other AP2 factors and chromatin remodeling machinery that represses gene expression. These findings add to our understanding of how *Toxoplasma* parasites balance replication and dormancy, revealing novel points of potential therapeutic intervention to disrupt this clinically relevant process.

## INTRODUCTION

Toxoplasma gondii, an obligate intracellular apicomplexan parasite of medical and veterinary interest, can infect almost all warm-blooded animals and is present in one-third of the human population. The complex life cycle of T. gondii involves multiple warm-blooded hosts and different developmental forms, including asexual and sexual stages. The sexual cycle takes place exclusively in the gut of felines, the definitive hosts for the life cycle, which consequently excrete infectious oocysts into the environment (1). The asexual phase of the life cycle is comprised of replicating tachyzoites and quiescent bradyzoites. Upon infection, the parasites increase their biomass and disseminate throughout the body as tachyzoites, which subsequently convert into latent bradyzoites that persist in brain, heart, and skeletal muscle tissue inside intracellular tissue cysts. The presence of bradyzoite cysts in the meat and organs of infected animals represents another major route of T. gondii transmission (2).

Despite the high prevalence in humans, acute toxoplasmosis is rarely seen and is most commonly observed as a reactivated infection in patients with HIV/AIDS or some other immunocompromised condition (3, 4). Congenital toxoplasmosis can also occur if tachyzoites traverse the placenta, which can lead to miscarriage or birth defects in humans, as well as abortion in livestock (5–7). Bradyzoite tissue cysts do not appear to be cleared effectively by the immune system of the host, nor are they targeted by the currently approved therapeutics (8–10). The formation of latent bradyzoite cysts is central to T. gondii pathogenesis and transmission, but the molecular mechanisms involved in stage conversion are incompletely understood (11, 12).

The hunt for T. gondii transcription factors that coordinate the reprogramming of the genome required for stage conversion has been challenging due to a striking lack of conventional master regulators in the parasite genome (13). In 2005, a new family of proteins that possess a DNA-binding domain related to the Apetala-2 transcription factors of plants was identified in apicomplexan parasites (14). These proteins are called ApiAP2 factors, and the T. gondii genome encodes 67 of these proteins (15–17), while the fellow apicomplexan parasite Plasmodium falciparum has 27 (14). In each parasite, multiple ApiAP2s have been linked to playing a role in regulating gene expression during specific developmental stages. For example, *Plasmodium* AP2-G regulates gametocyte development (18) and AP2-Sp2 regulates gene expression during the sporozoite stage (19). A number of ApiAP2s in T. gondii have been linked to tachyzoite stage conversion into bradyzoites (20). AP2IX-9 acts as a transcriptional repressor of bradyzoite genes and restricts commitment to development into *in vitro* bradyzoite tissue cysts (20). Like AP2IX-9, AP2IV-3 is upregulated during pH-induced *in vitro* bradyzoite differentiation, but acts as a transcriptional activator that likely competes to control bradyzoite gene expression with AP2IX-9 (21). Knocking out AP2IV-4 resulted in the expression of a subset of bradyzoite-specific proteins in replicating tachyzoites, which prevented tissue cyst formation in mice (22).

We previously determined that AP2IX-4 is a cell cycle-regulated factor expressed exclusively during division of tachyzoites and bradyzoites (23). Genetic knockout of AP2IX-4 had no discernible effect on tachyzoites, but reduced the frequency of tissue cyst formation *in vitro* and *in vivo* in mice (23). Transcriptional profiling showed that PruΔ*ap2IX-4* parasites display upregulation of a subset of bradyzoite genes over and above that seen in parental parasites exposed to alkaline stress, suggesting that it functions as a repressor of these genes. Despite the upregulation of several key bradyzoite genes, PruΔ*ap2IX-4* parasites showed a modest decrease in bradyzoite conversion in culture and in BALB/c mice.

To better understand the role of AP2IX-4, we purified the AP2IX-4 complex in this study, finding that it associates with the microrchidia (MORC) transcriptional repressor complex (24). We further interrogated the function of an AP2IX-4-interacting protein, AP2XII-2, which largely shares its cell cycle expression profile. In contrast to the dispensable AP2IX-4, tachyzoites lacking AP2XII-2 exhibit a fitness defect *in vitro*. Further experiments showed that AP2XII-2 is important for proper progression through the S-phase of the tachyzoite cell cycle and that its loss increases *in vitro* bradyzoite differentiation. These findings shed new light on the complex interplay between multiple AP2 factors regulating cell cycle and bradyzoite development in T. gondii.

## RESULTS

### Identification of proteins interacting with AP2IX-4.

**FIG 1:**
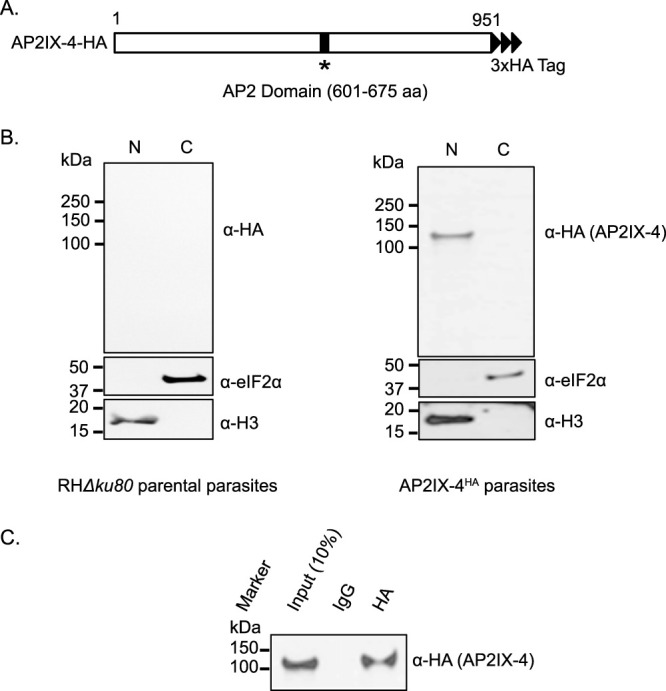
Endogenously tagged AP2IX-4^HA^ localizes to parasite nucleus. (A) Diagram of T. gondii AP2IX-4^HA^ protein. aa, amino acids. (B) Nuclear (N) and cytosolic (C) fractions of RHΔ*ku80* parental tachyzoites (negative control) and RHΔ*ku80* parasites expressing AP2IX-4^HA^ protein were generated for Western blot analysis using rat anti-HA antibodies. Each blot was reprobed with anti-TgIF2α as a cytosolic marker and anti-histone H3 as a nuclear marker. (C) Western blot of AP2IX-4 immunoprecipitated from purified intracellular RHΔ*ku80* tachyzoites via the use of Dynabeads coupled with mouse anti-HA antibodies. Western blots were probed using rat anti-HA antibody. Tagged AP2IX-4^HA^ migrates at the expected size of ∼104 kDa.

We previously identified a cell cycle-regulated ApiAP2 transcription factor called AP2IX-4 (T. gondii ME49_288950 [TGME49_288950]) that is expressed exclusively in dividing parasites (23). Genetic depletion of AP2IX-4 resulted in dysregulation of stage-specific gene regulation, which lowered the frequency of bradyzoite tissue cyst formation *in vitro* and *in vivo*. To better understand the role of AP2IX-4, we engineered RHΔ*ku80* tachyzoites to express endogenous AP2IX-4 protein with a C-terminal 3×HA (3× hemagglutinin) tag for use in immunoprecipitation experiments (AP2IX-4^HA^) (Fig. 1A). Western analysis of fractionated nuclear and cytosolic extracts revealed that AP2IX-4 is exclusively expressed in the parasite nucleus (Fig. 1B). Immunoprecipitation from the nuclear fraction using anti-HA purified a protein of the expected size of ∼104 kDa (Fig. 1C).

**FIG 2:**
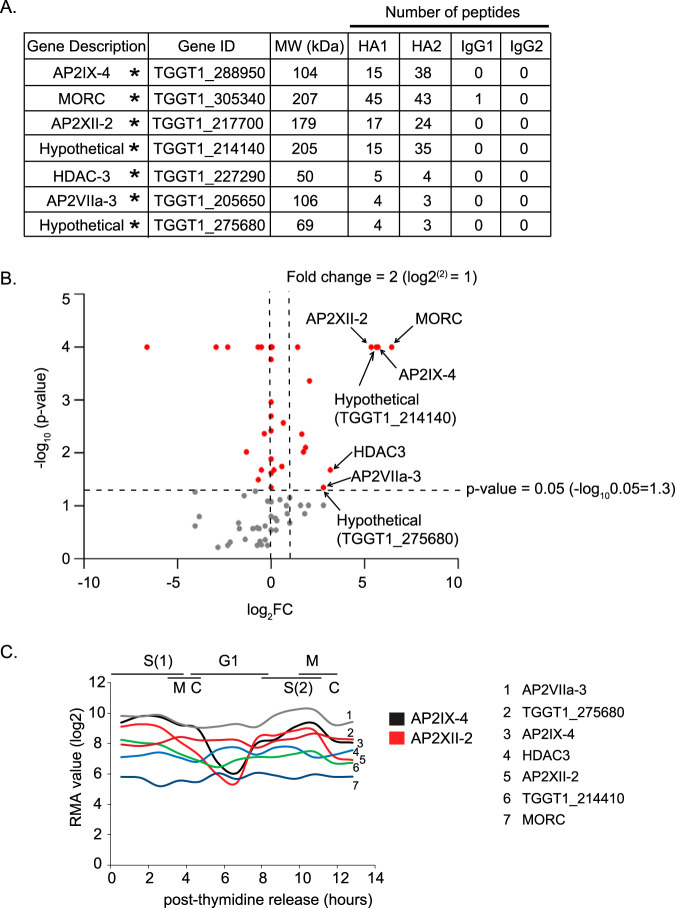
AP2IX-4 core complex in *Toxoplasma* tachyzoites. (A) Results of mass spectrometry analysis of endogenously tagged AP2IX-4^HA^ immunoprecipitate from tachyzoites, performed on intracellular parasites harvested from infected HFF cells. Two biological replicates were performed for AP2IX-4 immunoprecipitation experiments. Listed are proteins interacting with AP2IX-4 with probability of interaction as measured by a pSAINT value of 0.9 or above (see Table S1 for complete data set). Asterisks (*) denote proteins that were also identified in the MORC complex. ID, identifier; MW, molecular weight. (B) Volcano plot representing proteins copurified in the AP2IX-4^HA^ pulldown and identified by mass spectrometry, displaying statistical significance (*P* value) and fold change and each hit. The *x* axis plots the log_2_ fold change (HA/IgG), and the *y* axis demonstrates *P* values (statistical significance) in −log_10_. The horizontal dashed line indicates the *P* value (0.05) selected as the significance threshold. (C) Expression of transcripts for key proteins found in association with AP2IX-4 (source of data: ToxoDB.org). RMA, robust multiarray average; G1, G1 phase; S, synthesis phase; M, mitosis phase; C, cytokinesis phase.

Mass spectrometry and SAINT analysis of proteins coimmunoprecipitating with AP2IX-4HA included two additional ApiAP2 factors: AP2XII-2 (TGGT1_217700) and AP2VIIa-3 (TGGT1_205650) (Fig. 2A; see Table S1 in the supplemental material for complete data set). We also detected proteins interacting with AP2IX-4 that are associated with transcriptional repressive activity in tachyzoites: histone deacetylase 3 (HDAC3) (TGGT1_227290) and microrchidia, or MORC (TGGT1_305340, formerly CRC230), and two hypothetical proteins (TGGT1_214140 and TGGT1_275680) (Fig. 2A). Panel B of Fig. 2 shows a volcano plot generated with *P* values (−log_10_ *P* values) and fold change values (log_2_ FC) for the 136 protein hits detected from mass spectrometry. Together, the proteins complexed with AP2IX-4 overlap the recently elucidated microrchidia (MORC) repressor complex that has been implicated in silencing genes associated with the T. gondii sexual stages (24). Thus, our findings bolster the observation that multiple AP2 factors recruit MORC to regulate gene expression and provide an explanation as to why the loss of AP2IX-4 resulted in the dysregulation of genes (23).

10.1128/mSphere.00542-20.1 TABLE S1 Complete proteomics dataset for mass spectrometry analysis of AP2IX-4 immunoprecipitate. Download Table S1, XLS file, 0.05 MB. Copyright © 2020 Srivastava et al. 2020 Srivastava et al. This content is distributed under the terms of the Creative Commons Attribution 4.0 International license.

Panel C of Fig. 2 displays the mRNA expression profiles of AP2IX-4 and its interacting proteins (based on previously published data [22]). AP2IX-4 and AP2XII-2 have nearly parallel cell cycle-dependent expression patterns that reach their peaks during the S/M phase of the cell cycle before dropping in G_1_ phase. These data suggest that AP2IX-4 and AP2XII-2 operate during S/M phase. The mRNA levels of the other proteins pulled down with AP2IX-4 represented constitutive expression. Although it showed considerably lower peptide counts, AP2VIIa-3 is another AP2 factor that purified in the AP2IX-4 interactome that was also identified in the MORC complex (24). AP2VIIa-3 is not cell cycle regulated (Fig. 2C) and was not analyzed further in this study.

### Localization and interaction between AP2IX-4 and AP2XII-2.

**FIG 3:**
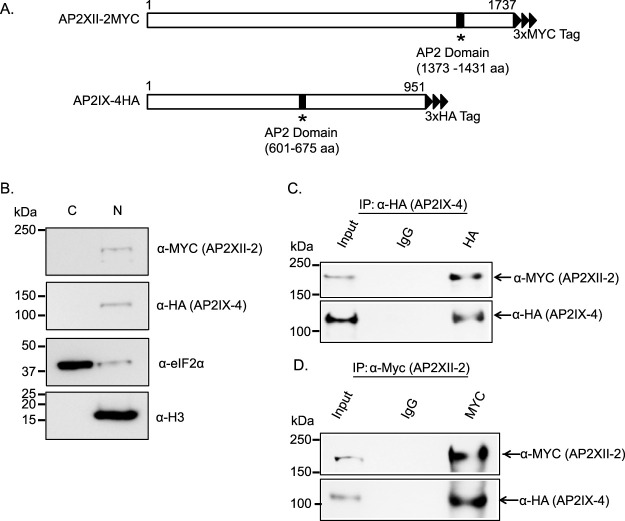
Interaction of AP2IX-4 and AP2XII-2. (A) Schematic representation of candidate interacting protein AP2XII-2 endogenously tagged with a c-MYC epitope. (B) Western blot of fractionated parasites showing that both AP2XII-2^MYC^ and AP2IX-4^HA^ localize to the parasite nucleus. The blot was reprobed with anti-TgIF2α and anti-histone H3 as cytosolic and nuclear markers, respectively. (C) Coimmunoprecipitation (Co-IP) confirmed the interaction between AP2XII-2^MYC^ and AP2IX-4^HA^ in tachyzoites. (Top panel) IP was performed with anti-HA to pull down AP2IX-4^HA^; probing with anti-MYC detected AP2XII-2^MYC^ in the IP. (Bottom panel) The membrane was reprobed with anti-HA. (D) Reciprocal Co-IP further confirmed the interaction; in this case, the IP was performed with anti-MYC to pull down AP2XII-2^MYC^. The top panel shows that probing performed with anti-MYC detected AP2XII-2^MYC^. The bottom panel shows the results of reprobing with anti-HA, which detected AP2IX-4^HA^ in the IP.

The single exon of the AP2XII-2 gene encodes a 195-kDa protein containing 1,431 amino acids and a single AP2 domain proximal to the C terminus (Fig. 3A). To validate the protein-protein interaction and study AP2XII-2 further, we endogenously tagged the C terminus of AP2XII-2 with 3×MYC in the RHΔ*ku80* parasites expressing AP2IX-4^HA^. Immunoblots of these dual-tagged parasites confirmed the presence of both in the nuclear fraction (Fig. 3B). We performed reciprocal coimmunoprecipitations of AP2IX-4^HA^ and AP2XII-2^MYC^ followed by Western blotting, which validated their interaction in intracellular tachyzoites (Fig. 3C and D).

**FIG 4:**
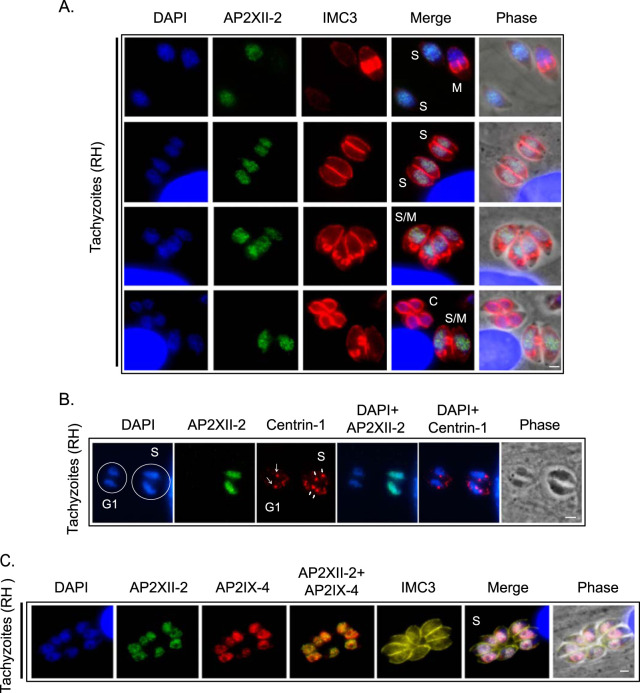
Immunofluorescence assay of AP2IX-4 and AP2XII-2 during cell cycle. (A) RHΔ*ku80* (RH) parasites expressing endogenously tagged AP2XII-2^MYC^ were probed with antibodies to c-MYC (green) and IMC3 (red), the latter of which helps detect budding daughter parasites. AP2XII-2^MYC^ was detectable in the S and S/M phases but disappeared before the onset of cytokinesis (C). DAPI (blue) was used as a nuclear stain. The four rows show parasites in different stages of the cell cycle. Scale bar = 2 μm. (B) To more closely examine the expression of AP2XII-2^MYC^ during cell cycle, an IFA was performed using antibodies to c-MYC (green) and Centrin-1 (red). Parasites in G_1_ contain single centrosomes, whereas those in S-phase are duplicated. Arrows indicate centrosomes. Scale bar = 2 μm. (C) Triple-labeled IFA to determine cell cycle expression patterns for each AP2 factor. AP2XII-2^MYC^ (green) and AP2IX-4^HA^ (red) colocalized in S-phase. IMC3 (yellow) and larger nuclei (DAPI, blue) identify parasites in S-phase. Scale bar = 2 μm.

We next monitored the pattern of expression for AP2XII-2^MYC^ by immunofluorescence assay (IFA) for comparison to what we had observed previously for AP2IX-4 (23). To delineate the cell cycle expression profile of AP2XII-2, we costained with established cell cycle markers, including Inner membrane complex-3 (IMC3) and T. gondii Centrin-1 (TgCentrin-1) (Fig. 4A) (25, 26). Results show that AP2XII-2^MYC^ protein was expressed in the second half of the cell cycle prior to the start of budding and continued until the buds were mature, in agreement with mRNA profiling results (Fig. 2C). This protein expression pattern is consistent with a cell cycle timing that begins in S phase and continues into mitosis, with the factor downregulated after nuclear division (very early G_1_ period). Expression of AP2XII-2 diminishes before the onset of C-phase (cytokinesis); this is in contrast to AP2IX-4, which can be detected through to the end of cytokinesis (23). By costaining with TgCentrin-1, we identified distinct parasite vacuoles in either G_1_ or S-phase of the cell cycle in the same field; note that AP2XII-2 is detected in parasites only during S-phase and not in G_1_ (Fig. 4B). By staining with anti-MYC, anti-HA, and anti-IMC3, we were able to show that both AP2 proteins are coexpressed during S-phase of the cell cycle (Fig. 4C). Altogether, these data support an intimate interaction between AP2IX-4 and AP2XII-2 during parasite division.

### Conditional knockdown of AP2XII-2 slows parasite replication.

To further investigate the role of AP2XII-2 in T. gondii, we attempted to genetically ablate the endogenous AP2XII-2 gene. Both conventional allelic replacement using homologous recombination and CRISPR/Cas9 approaches yielded no viable parasites, suggesting that AP2XII-2 is essential in tachyzoites. A genome-wide CRISPR survey assigned this AP2 a score of −1.16, suggestive that the loss of AP2XII-2 compromises fitness (22).

**FIG 5:**
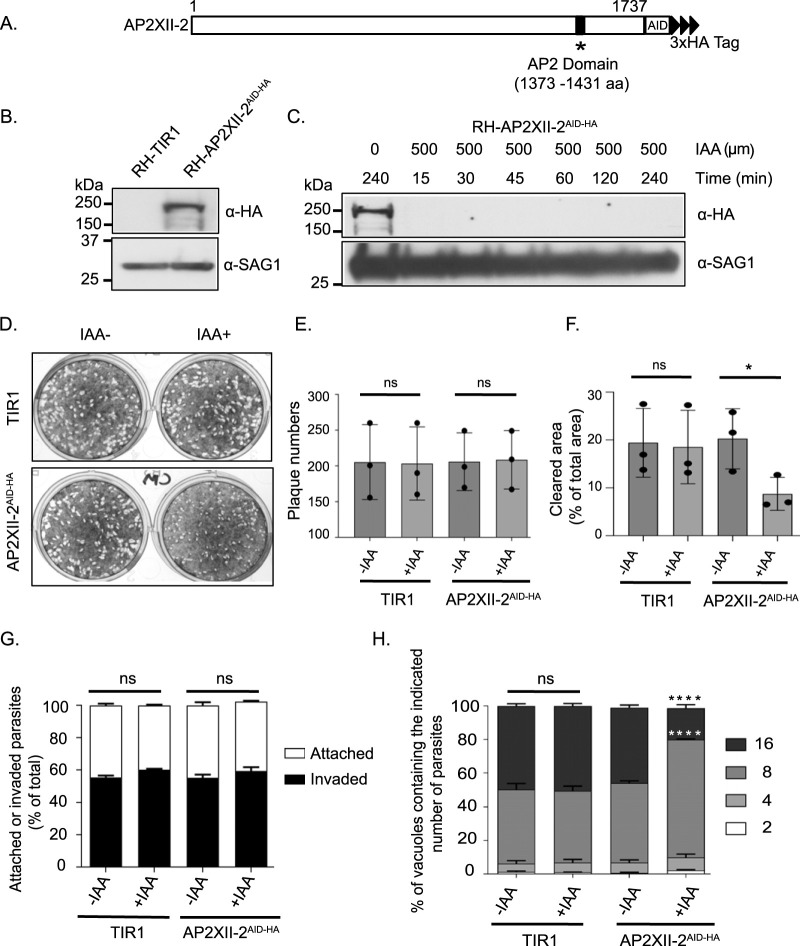
Conditional knockdown of AP2XII-2. (A) Schematic representation of AP2XII-2 endogenously tagged with AID-HA at the C terminus. (B) Western blot showing expression of AP2XII-2^AID-HA^ in RH-TIR1 parental parasites at the expected size (∼250 kDa). SAG1 serves as a loading control. (C) Western blot probed with anti-HA to monitor AP2XII-2^AID-HA^ protein levels over a time course after addition of 500 μM IAA. SAG1 was used as a loading control. (D) Representative plaque assay image of AP2XII-2^AID-HA^ parasites treated with 500 μM IAA or vehicle (ethanol [EtOH]) for 6 days, showing the presence of smaller plaque sizes when AP2XII-2 was depleted. (E) Number of plaques generated for parental parasite (TIR1) and AP2XII-2^AID-HA^ in the presence (+) or absence (-) of IAA 6 days postinfection. Each dot represents average of each experiment (± standard deviation [SD], *n* = 3). Unpaired Student's *t* test was performed for analysis; ns, not significant. (F) The degree of host cell monolayer lysis was quantified for the plaque assays. Each dot represents the average of results from each experiment (± SD, *n* = 3). Unpaired Student's *t* test was performed for analysis. ns, not significant; *, *P* < 0.05. (G) AP2XII-2^AID-HA^ parasites were grown in the presence of 500 μM IAA or vehicle (EtOH) for 24 h, forcibly lysed from host cells, and then allowed to invade a new HFF monolayer for 30 min for attachment and invasion assay. The data represent means ± SD (*n* = 3). Data were analyzed using two-way analysis of variance (ANOVA) and Tukey's multiple-comparison test. ns = not significant. (H) Doubling assays were performed to assess replication of AP2XII-2^AID-HA^ parasites in the presence of 500 μM IAA or vehicle (EtOH). Parasites were counted from 100 random vacuoles following 24 h of infection. The numbers of parasites were plotted as percentages of the total number of parasite vacuoles. The values represent means ± SD (*n* = 3). Two-way ANOVA with Tukey's multiple-comparison test was performed. ****, *P* < 0.0001; ns = not significant.

We therefore pursued an indole-3-acetic acid (IAA)-inducible degradation (AID) strategy that would allow a conditional knockdown of AP2XII-2 protein through the addition of IAA (27). We endogenously tagged AP2XII-2 with a C-terminal AID-3×HA tag in RH-TIR1-3×Flag Δ*ku80* parasites (27) (Fig. 5A and B). A clone was isolated in which AP2XII-2^AID-HA^ protein was undetectable within 15 min after inclusion of IAA in the culture medium (Fig. 5C). To determine the role of AP2XII-2 in parasite viability, we performed a standard plaque assay in the presence of IAA. The loss of AP2XII-2 resulted in smaller plaque sizes than were seen with the parental parasites, although there was no change in plaque number (Fig. 5D to F). These results suggest a defect in parasite replication rather than invasion. To confirm this idea, we cultured parasites in 500 μM IAA for 24 h and then subjected them to syringe lysis and filter purification for an attachment and invasion assay (27). We found that AP2XII-2 depletion had no effect on invasion or attachment of parasites (Fig. 5G). We also conducted a parasite counting assay in the presence of IAA or vehicle (ethanol [EtOH]), finding that replication rates were significantly lower without AP2XII-2 (Fig. 5H). AP2XII-2^AID-HA^ parasites treated with IAA for 24 h contained more vacuoles with only 8 parasites and fewer vacuoles with 16 parasites.

### Loss of AP2XII-2 delays S-phase and increases bradyzoite differentiation.

**FIG 6:**
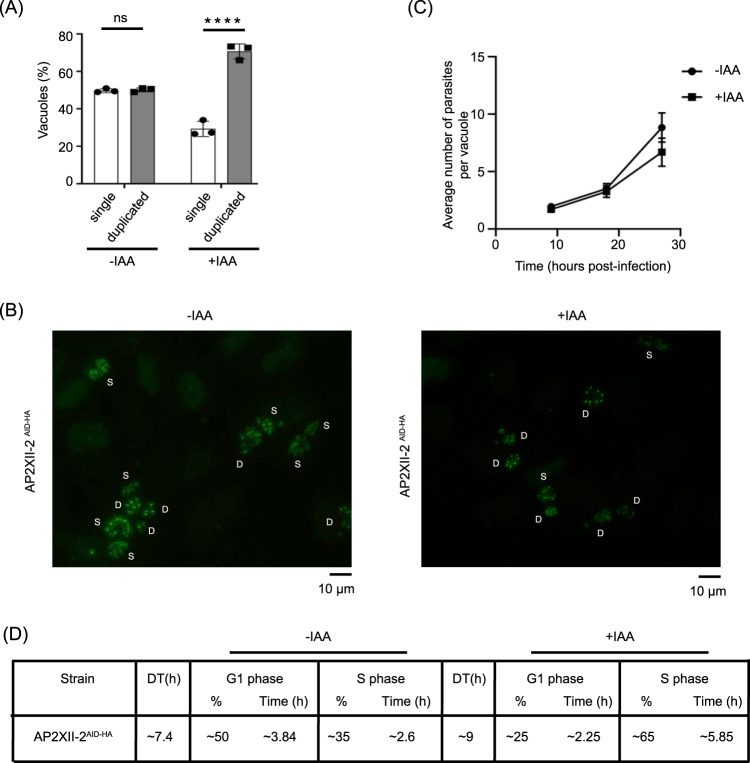
AP2XII-2 depletion results in a delay in S-phase of cell cycle. (A) A total of 100 parasite vacuoles in 10 random fields were counted for single or duplicated centrosomes in 3 biological replicates. Tukey's multiple-comparison test was performed with two-way ANOVA. Error bars represent standard deviations of the means; ****, *P* < 0.0001; ns = not significant. (B) Representative IFAs of AP2XII-2^AID-HA^ parasites with or without IAA showing single (S) and duplicated (D) centrosomes as stained by anti-Centrin-1 (green). Scale bar =10 μm. (C) The number of parasites per vacuole was counted at each indicated time point for 100 randomly chosen vacuoles to calculate doubling time of AP2XII-2^AID-HA^ parasites with or without IAA. Error bars represent mean ± SD (*n* = 3). The exponential growth equation was used to calculate the doubling time in GraphPad Prism software as described previously (29). (D) The percentage of AP2XII-2^AID-HA^ parasites (with [+] or without [-] IAA) in various stages of the cell cycle was determined as described in the text. Doubling times (DT) in hours determined as described for panel C are also shown.

We further examined the replication defect seen in AP2XII-2-depleted parasites by centrosome counting. Normally, in an asynchronous parasite population in culture, 50% of parasites are in G_1_ phase (single centrosome) and 50% of parasites are in S-phase undergoing DNA replication (duplicated centrosome) (28). Without IAA, the AP2XII-2^AID-HA^ parasites showed a 50/50 split of single and duplicated centrosomes within the population; in contrast, when AP2XII-2^AID-HA^ parasites were treated with IAA, ∼75% of the population exhibited duplicated centrosomes, supporting a delay in S-phase of the cell cycle (Fig. 6A and B).

To further characterize the cell cycle defect, we compared the lengths of the S-phase in AP2XII-2^AID-HA^ parasites with or without IAA by calculating doubling time from average vacuole size as described previously (29). The parasites expressing AP2XII-2 had a doubling time of 7.4 h, whereas those depleted of AP2XII-2 showed an increase in the doubling time to 9.0 h (Fig. 6C). We then determined the percentages of parasites in the different cell cycle stages using IFAs probed with markers for the centrosome (anti-Centrin-1) and daughter cell budding (anti-IMC3) as described previously by Szatanek et al. (30). Parasites containing a single centrosome without daughters are in G_1_, parasites with duplicated centrosomes without daughters are in S phase, and parasites with duplicated centrosomes and budding daughters are undergoing cytokinesis (C). In the parasites expressing AP2XII-2, approximately 50% were in G_1_, 35% were in S, and 14% were in C. In parasites depleted of AP2XII-2, this ratio was approximately 25% (G_1_), 65% (S), and 10% (C). We calculated the duration of G_1_ and S phase based on the doubling times and percentages of parasites in different stages of the cell cycle. Results show that the length of S-phase in parasites lacking AP2XII-2 was ∼5.85 h compared to ∼2.6 h when AP2XII-2 was expressed (Fig. 6D).

**FIG 7:**
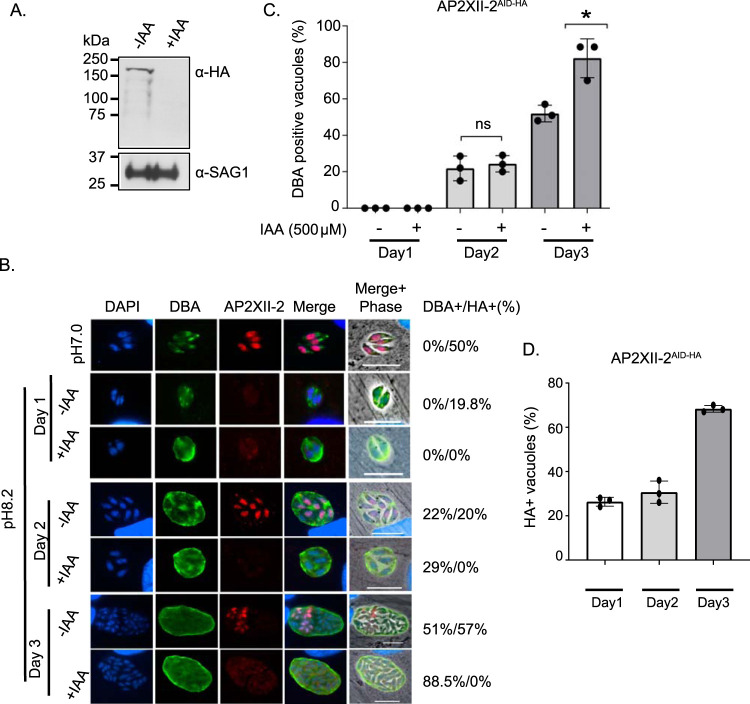
AP2XII-2 depletion leads to increased bradyzoite differentiation. (A) Western blot with anti-HA confirms the depletion of AP2XII-2^AID-HA^ in type II ME49-TIR1 parasites. SAG1 was used as a loading control. Parasites were treated with 500 μM IAA or vehicle (EtOH) for 24 h. (B) Type II AP2XII-2^AID-HA^ tachyzoites were induced to differentiation with alkaline pH 8.2 for 3 days in the presence of 500 μM IAA or vehicle (EtOH). Differentiating parasites were stained with anti-HA (red) and *Dolichos* lectin (DBA, green) to visualize tissue cyst wall. DAPI (blue) was included to highlight nuclei. Scale bar = 10 μm. (C and D) Graphical depiction of data from panel B. A total of 100 random vacuoles were counted for *Dolichos* (DBA)-positive (C) and HA-positive (D) staining, shown to the right of the IFA panels. *n* = 3 for control and 500 μM IAA-treated parasites. Error bars represent standard errors of the means; ns = not significant; *, *P* < 0.05 (unpaired two-tailed Student's *t* test).

It was shown previously that the initiation of bradyzoite differentiation and tissue cyst formation requires a slowing in DNA replication, delayed S-phase, and lengthening of G_1_ phase (31, 32). Given the delay in S-phase produced by depletion of AP2XII-2, we examined whether there is also an increased frequency of bradyzoite development. To do so, we endogenously tagged AP2XII-2 with AID-HA in type II ME49-TIR1 parasites (33). A clone was isolated in which AP2XII-2^AID-HA^ protein was undetectable after inclusion of IAA in the culture medium (Fig. 7A). The deficiency in plaque size shown by ME49 AP2XII-2^AID-HA^ parasites treated with IAA was the same as that shown by their RH counterparts (data not shown). We then examined the frequency of tissue cyst formation of ME49 AP2XII-2^AID-HA^ parasites incubated under conditions of alkaline stress with or without IAA (Fig. 7B). After 3 days in alkaline media without IAA, ∼50% of parasite vacuoles were staining positive with the cyst wall marker *Dolichos*. However, AP2XII-2^AID-HA^ parasites maintained in media with IAA displayed ∼88% conversion into bradyzoite cysts (Fig. 7B and C). No overt changes in the size or morphology of the cysts formed were evident. Type II ME49-TIR1 parasites did not show any difference in differentiation when incubated in the presence of IAA under alkaline stress conditions (data not shown), as previously reported (33). It should be noted that IAA did not enhance bradyzoite formation at day 1 or day 2, supporting the idea that IAA is not a contributing factor in the increased frequency of tissue cyst formation.

Consistent with the restricted cell cycle expression of AP2XII-2 in tachyzoites, this factor was detected in replicating parasites only following alkaline medium stress (Fig. 7B), an expression pattern also shown by its interacting AP2, AP2IX-4 (23). Also similarly to AP2IX-4, AP2XII-2 expression levels decreased during the initial stages of bradyzoite differentiation and then increased again. Upon addition of alkaline stress, AP2XII-2 expression levels dropped from 50% to 20% within the parasite population, but by day 3, AP2XII-2 was present in ∼55% of bradyzoite cysts (Fig. 7D).

Together, these results indicate that AP2XII-2 is an important contributor to timely progression through S-phase of the cell cycle and that its loss slows tachyzoite replication and promotes bradyzoite differentiation.

## DISCUSSION

In the present work, we report that AP2IX-4 can associate with the T. gondii MORC transcriptional repressor complex during its expression in S/M phases of the tachyzoite cell cycle. We also characterized a previously undescribed ApiAP2 interacting with AP2IX-4, AP2XII-2, which knockdown experiments revealed to be important for proper progression through S-phase.

There is evidence that commitment to differentiation may occur during the S/M phase of the tachyzoite cell cycle (34); it requires transient slowing of growth (35), delayed S-phase, and lengthening of the G_1_ phase (32). Bradyzoite cyst formation represents a complex developmental pathway that requires a cascade of events in which multiple ApiAP2 transcription factors appear to be involved (11). Research is just beginning to reveal the sophistication of the ApiAP2 network, which possesses both transcriptional repressors and activators. AP2IX-9, AP2IV-4, and AP2IX-4 repress transcription of bradyzoite genes and impair tissue cyst formation (20–23), whereas AP2IV-3 appears to activate transcription (21). Given their evolutionary origin, some members of the ApiAP2 family may exhibit features found in the plant kingdom, which has AP2 domain proteins classified as active and passive transcription factors (36). Passive repressors may compete with activators to dampen transcription, allowing a fine-tuning of gene expression (36). In addition to ApiAP2s, a “master regulator” transcription factor termed BFD1 (Bradyzoite Formation Deficient-1), which harbors a myb-like DNA-binding domain, has recently been described that is essential for tissue cyst formation (37). Both BFD1 and MORC bind the promoter of AP2IX-9, as well as other AP2 factors; MORC also associates with additional AP2 factors, many of which have yet to be characterized. How the various AP2 factors interplay with these other transcriptional regulators to affect gene expression remains an important focus for future investigation.

Our report also adds to the growing evidence indicating that at least some ApiAP2s coordinate transcriptional regulatory activities through interactions with chromatin remodeling machinery. We recently reported that the essential histone acetyltransferase (HAT) GCN5b operates in a multisubunit complex with at least two ApiAP2 factors, AP2IX-7 and AP2XII-4, to activate gene expression (38). In the current study, we found that AP2IX-4 and APXII-2 interact with the recently described MORC complex, which contains transcriptional repressive proteins, including histone deacetylase HDAC3 (24). Identifying AP2IX-4 as an interactor with MORC validates our previous observation that loss of AP2IX-4 results in upregulation of a subset of bradyzoite genes (23). It is likely that other ApiAP2s acting as repressors or activators mediate these regulatory events through collaboration with HDACs or HATs, respectively.

AP2IX-4 is a cell cycle-regulated factor with peak expression in S/M phase (23). The AP2IX-4 complex that we isolated contained two additional uncharacterized ApiAP2s, AP2XII-2 and AP2VIIa-3. The AP2XII-2 cell cycle expression pattern is highly similar to that of AP2IX-4, which we confirmed by IFA after tagging the endogenous protein. Like AP2IX-4, AP2XII-2 is a nuclear protein with peak expression during the S/M phase. However, AP2XII-2 expression decreases before the onset of C-phase whereas AP2IX-4 expression remains through cytokinesis. These findings suggest that the two AP2s may collaborate during tachyzoite division but that AP2IX-4 may have another, independent role toward the end of the process. Curiously, AP2IX-4 is dispensable in tachyzoites but loss of its interacting partner AP2XII-2 impairs replication due to a delay in S-phase, which likely explains the enhanced bradyzoite differentiation seen when AP2XII-2 is knocked down. AP2XII-2 may be able to compensate for the loss of AP2IX-4, but AP2IX-4 cannot replace the function of AP2XII-2. We noted that knockdown of AP2XII-2 did not affect AP2IX-4 transcript levels (data not shown), but potential changes in protein levels could not be assessed with the reagents in hand. It is also possible that AP2XII-2 has an additional function in the cell cycle that is independent of AP2IX-4. The precise role of AP2XII-2 in facilitating progress through S-phase is an important subject for future investigation. The roles performed by these two AP2 factors are likely to diverge under stress conditions or during bradyzoite development as knockout of AP2IX-4 impaired *in vitro* differentiation whereas knockdown of AP2XII-2 enhanced it.

As we observed for AP2IX-4 (23), the expression levels of AP2XII-2 protein dropped during the early stages of bradyzoite differentiation. AP2XII-2 was detectable in ∼20% of bradyzoite cysts at day 1 and day 2 postinduction but was present in >55% of bradyzoite cysts by day 3 (Fig. 7B). Since the S/M period becomes more pronounced during bradyzoite differentiation, the increase in AP2XII-2-positive parasite numbers further supports the idea of a defined cell cycle context for commitment to bradyzoite differentiation. We found previously that AP2IX-4 is present only in dividing parasites within cysts, and this is likely to be the case for AP2XII-2. We suspect that these *in vitro* bradyzoites are immature and still undergoing some level of cell division, which requires these two ApiAP2s for proper progression through the cell cycle. Our findings underscore the links between cell cycle progression, parasite division, and the commitment to bradyzoite differentiation and bolster the concept that cell cycle-regulated ApiAP2s such as AP2IX-4 and AP2XII-2 work with chromatin remodeling machinery like MORC to regulate gene expression relevant to cell cycle progression.

## MATERIALS AND METHODS

### Parasite culture and transfection.

The parasite strains used for this study included RHΔ*hxgprt*Δ*ku80* (39), RHΔ*hxgprt*Δ*ku80*:*AP2IX-4^HA^* (23), and RH and ME49 parasites engineered to express TIR1 for IAA-mediated protein degradation (27, 33).

Parasites were cultured in human foreskin fibroblast (HFF) cells in Dulbecco’s modified Eagle medium supplemented with 1% fetal bovine serum, 100 unit/ml penicillin, and 100 μg/ml streptomycin. Transfection of parasites was performed as previously described using a 4D-Nucleofector system (Lonza) and selection for drug resistance 24 h posttransfection (40–42). Transfected parasites were selected in either 1 μM pyrimethamine or 25 μg/ml mycophenolic acid plus 50 μg/ml xanthine as described previously (43, 44).

### Generation of parasites expressing endogenously tagged AP2IX-4^HA^ and AP2XII-2^MYC^.

RHΔ*hxgprt*Δ*ku80*:*AP2IX-4^HA^* (23) parasites were genetically modified to express endogenous AP2XII-2 tagged with MYC at the C terminus. Modification of the endogenous locus of AP2XII-2 was performed by a genetic knock-in approach using plasmid pLIC-3xMYC-HXGPRT (39). Primers (Fw, 5′-ATCCAATTTAATTAATCTTCTGTGTCCCGGTGG-3′; Rv, 5′-TCCAATTTTAATTAAGCCACTGAGTGGTGAAACA-3′) were used to amplify 1,975 bp of the AP2XII-2 gene, which was then cloned into the PacI site of pLIC-3xMYC-HXGPRT plasmid. The resulting construct was linearized and transfected into RHΔ*hxgprt*Δ*ku80*:*AP2IX-4^HA^* parasites. Transfected parasites were selected in 25 μg/ml mycophenolic acid plus 50 μg/ml xanthine, and independent clones were isolated by limiting dilution.

### Generation of parasites expressing endogenously tagged AP2XII-2^AID-HA^.

To generate type I and type II conditional knockdown strains, specific primers (Fw, 5′-ACCGGGCCCGCTAGCTCTTCTGTGTCCCGGTGG-3′; Rv, 5′-CGAGCCCTTGCTAGCGCCACTGAGTGGTGAAACA-3′) were used to amplify 1,975 bp of the AP2XII-2 gene for cloning into the NheI site of pLIC-AID-3xHA-DHFR plasmid. The construct was linearized and transfected into either RH or ME49-TIR1-expressing parasites (27, 31). Parasites were selected in 1 μM pyrimethamine, and single clones were isolated by limiting dilution. Parasites were treated with 500 μM IAA (Sigma-Aldrich) or vehicle (EtOH) for the indicated period of time to induce degradation of AID-tagged protein.

### Parasite growth assays.

For plaque assays, 500 freshly syringe-lysed intracellular parasites were allowed to invade a confluent monolayer of HFF cells grown in 12-well plates in 500 μM IAA or vehicle (EtOH). Six days postinfection, the infected monolayers were stained with crystal violet stain to determine host cell lysis as previously described (45).

For doubling assays, purified intracellular parasites were allowed to invade confluent HFF cells for 30 min. After invasion, infected HFF cells were gently washed with culture medium to remove extracellular tachyzoites and fresh medium was added. After 24 h, infected monolayers were fixed with 4% paraformaldehyde for 10 min at room temperature, incubated in blocking buffer for 30 min, and then stained with anti-SAG1 antibody (Invitrogen) for 1 h at room temperature. The fixed cells were then washed with phosphate-buffered saline (PBS) and incubated with secondary goat anti-mouse Alexa Fluor 488 (Invitrogen) for 1 h at room temperature. Cells were mounted with ProLong gold antifade mounting solution (Invitrogen) containing DAPI (4′,6-diamidino-2-phenylindole). The number of parasites in 100 randomly selected vacuoles was counted at the designated time point (46).

### Invasion and attachment assays.

Invasion and attachment assays were performed as previously described (27, 47). Briefly, parasites were allowed to grow in 500 μM IAA or vehicle (EtOH) for 24 h and then purified from host cells via syringe lysis. The parasites were allowed to invade confluent HFF cells for 30 min prior to fixation with 4% paraformaldehyde (Sigma). Attached parasites were labeled with mouse anti-SAG1 (Thermo Fisher) and then subjected to permeabilization in 0.1% Triton X-100. Next, parasites were stained with anti-IMC3 antibody (a gift from Marc-Jan Gubbels) followed by anti-mouse Alexa Fluor 488 (Invitrogen) and anti-rat Alexa Fluor 594 (Invitrogen). Ten random fields were imaged, and the number of attached and invaded parasites was counted. Primary antibody dilutions were used as follows: mouse anti-SAG1 (Thermo Fisher) 1:1,000 and rat anti-IMC3 1:2,000. Secondary Alexa Fluor antibodies were used at 1:1,000 dilution.

### Western blotting.

Confluent HFF cells infected with tachyzoites were subjected to syringe lysis 24 h postinfection and filtered, followed by centrifugation. Parasite pellets were resuspended in NuPAGE lysis buffer and boiled for 5 min. Parasite lysates (80 μg) were separated on a 4% to 12% Tris-acetate polyacrylamide gradient gel (Invitrogen) and transferred onto polyvinylidene difluoride (PVDF) membranes. Western blotting was performed with the designated antibodies, and the blots were developed with SuperSignal West Femto sensitivity substrate (Pierce).

Primary antibody dilutions were used as follows: rat anti-HA antibody (Roche) 1:2,000; mouse anti-MYC (Cell Signaling) 1:5,000; rabbit anti-MYC (Thermo) 1:5,000; rabbit anti-H3 (Sigma) 1:5,000; rabbit eukaryotic initiation factor 2α (eIF2α) 1:20,000 (48); mouse anti-Sag1 (Thermo Fisher) 1:5,000. Secondary horseradish peroxidase (HRP)-conjugated antibody dilutions were used as follows: goat anti-rat (GE Healthcare) 1:2,000; goat anti-mouse (Sigma) 1:5,000; goat anti-rabbit (Sigma) 1:5,000.

### Immunofluorescence assays (IFA).

Parasites growing in confluent HFF cells were fixed in 4% paraformaldehyde for 10 min followed by washing in PBS three times. Fixed cells were incubated for 1 h at room temperature in blocking buffer containing 3% bovine serum albumin (BSA) and 0.2% Triton X-100. The cells were incubated with the designated antibodies in blocking buffer at 4°C overnight followed by washing in PBS. The cells were then incubated with secondary antibodies coupled to Alexa Fluor 488/594/568/647 at room temperature for 1 h. The cells were finally washed with PBS and mounted with ProLong gold antifade mounting solution (Invitrogen) containing DAPI (Invitrogen) and then visualized using a Nikon Eclipse E100080i microscope. Images were captured with a Hamamatsu C4742‐95 charge-coupled-device (CCD) camera. Nikon NIS element software was used to analyze and capture images. Primary antibody dilutions were used as follows: rabbit anti-HA (Cell Signaling) 1:1,000, rat anti-HA (Roche) 1:1,000, mouse anti-MYC (Cell Signaling) 1:1,000, *Toxoplasma* anti-Centrin-1 (Kerafast Inc.) 1:2,000, rat anti-IMC3 (supplied by Marc-Jan Gubbels) 1:2,000. For the visualization of bradyzoite tissue cyst walls, fluorescein isothiocyanate (FITC)-conjugated Dolichos biflorus lectin (Vector Laboratories) was used at a 1:500 dilution for 1 h at room temperature as previously described (23).

### Bradyzoite differentiation assay.

*In vitro* bradyzoite differentiation experiments were performed as described elsewhere (20, 49, 50). Briefly, parasites were allowed to invade a confluent monolayer of HFF cells grown in 12-well plates. After 2 h postinvasion, tachyzoite medium (pH 7.0) was replaced with bradyzoite differentiation medium (pH 8.2), and parasites were grown in the presence of IAA (500 μM) or vehicle (EtOH) for the indicated time periods (20, 49, 50). The medium was changed every 24 h to maintain pH for parasites undergoing differentiation, with fresh IAA (500 μM) or vehicle (EtOH) added. A hundred random fields were selected in a blind under phase contrast, and then the numbers of parasite vacuoles were counted for DBA and HA positivity by changing the corresponding channels. If any parasites within the vacuole contained HA signal, the result was scored as positive.

### Subcellular fractionation.

Purified parasite pellets were resuspended in 1 ml low-salt buffer (50 mM HEPES-NaOH [pH 7.5], 20% glycerol, 10 mM NaCl, 0.1% NP-40) and incubated at 4°C for 10 min followed by centrifugation at 2,500 × *g* for 15 min. The supernatant was collected as the cytoplasmic fraction. The pellet containing the nuclei was resuspended in high-salt extraction buffer (50 mM HEPES-NaOH [pH 7.5], 20% glycerol, 420 mM NaCl, 0.4% NP-40) for 10 min at 4°C with intermittent stirring followed by brief sonication (5 rounds, 10 s each). Following centrifugation at 13,000 × *g* for 30 min, the cleared nuclear fraction supernatant was collected. All buffers were supplemented with cOmplete Mini, EDTA-free protease inhibitor cocktail (Roche, catalog no. 11 836 170 001).

### Purification of the AP2IX-4 complex.

RHΔ*hxgprt* parasites endogenously expressing AP2IX-4^HA^ or AP2IX-4^HA^/AP2XII-2^MYC^ were used to pull down the core AP2IX-4 complex. Large-scale tachyzoite cultures were grown for 24 h in HFF cells, followed by scraping the infected monolayers and syringe lysis through a 25-gauge needle to isolate free tachyzoites. Parasites were further purified through a 3-μm-pore-size membrane filter and centrifuged at 4°C for 10 min at 700 × *g*. The parasite pellets were lysed in low-salt buffer (50 mM HEPES-NaOH [pH 7.5], 20% glycerol, 10 mM NaCl, 0.1% NP-40) for 10 min on ice followed by centrifugation at 2,500 × *g* for 10 min to isolate soluble cytosolic and nuclear pellet fractions. The nuclear pellet was washed twice with low-salt buffer and then treated with MNase (Thermo Fisher Scientific) in the presence of 1 mM CaCl_2_ for 10 min at 37°C followed by addition of EDTA to stop MNase digestion. The samples were centrifuged again to isolate MNAse-soluble chromatin fractions and MNAse-insoluble pellet fractions. The MNAse-insoluble pellet fractions were dissolved in high-salt buffer (50 mM HEPES-NaOH [pH 7.5], 20% glycerol, 420 mM NaCl, 0.4% NP-40) for 30 min with rocking at 4°C. Finally, the high-salt fraction was isolated by centrifugation. Soluble fractions were combined for immunoprecipitation with anti-HA or anti-MYC antibodies coupled with magnetic Dynabeads per the instructions of the manufacturer (Thermo Fisher). Immunoprecipitated proteins on the beads were boiled in SDS sample buffer for 5 min and processed for Western blotting or proteomics analysis. All buffers used in the protocol were supplemented with Roche cOmplete Mini EDTA-free protease inhibitor cocktail and 1 mM phenylmethylsulfonyl fluoride (PMSF).

### Mass spectrometry of the AP2IX-4 complex.

Sample preparation and mass spectrometry analysis were performed as previously described (38). Briefly, immunoprecipitated samples on Dynabeads were resuspended in 8 M urea for denaturation, reduced, and alkylated. The alkylated samples were digested with endoproteinase LysC and trypsin gold (Promega) at 37°C overnight. The digested peptides were purified with a spin column (Pierce), followed by injection into a C_18_ reversed-phase trap column (3 μm pore size). The peptides were then separated on an EASY-Spray 15-cm reversed-phase analytical column (Thermo Fisher) (2 μm pore size). Peptides were separated on acetonitrile (2% to 25% gradient for 90 min) in front of a Velos Pro Orbitrap mass spectrometer in data-dependent acquisition mode. Liquid chromatography/mass spectrometry (LC/MS) data were obtained using Proteome discoverer software and searched against the ToxoDB database. The list of 136 total proteins identified was generated with Scaffold proteome software. Bioinformatics analysis was performed as described previously (17). Briefly, the filters used for hit selection from Scaffold software were as follows: minimum number of peptides, 2; protein threshold, 99.9%; peptide threshold, 95%; false-discovery rate (FDR), 0%. SAINT (Significance Analysis of INTeractome) analysis of interactome data was performed as previously described (38).

### Statistical analysis.

GraphPad Prism software was used for statistical analyses. SAINTexpress software was used for SAINT analysis.
